# Sulfadoxine-Pyrimethamine–Based Combinations for Malaria: A Randomised Blinded Trial to Compare Efficacy, Safety and Selection of Resistance in Malawi

**DOI:** 10.1371/journal.pone.0001578

**Published:** 2008-02-13

**Authors:** David J. Bell, Suzgo K. Nyirongo, Mavuto Mukaka, Ed E. Zijlstra, Christopher V. Plowe, Malcolm E. Molyneux, Steve A. Ward, Peter A. Winstanley

**Affiliations:** 1 Department of Molecular and Biochemical Parasitology, Liverpool School of Tropical Medicine, Liverpool, United Kingdom; 2 Malawi-Liverpool-Wellcome Trust Clinical Research Programme, Blantyre, Malawi; 3 Department of Medicine, College of Medicine, University of Malawi, Blantyre, Malawi; 4 Center for Vaccine Development, University of Maryland School of Medicine, Baltimore, Maryland, United States of America; 5 School of Clinical Sciences, University of Liverpool, Liverpool, United Kingdom; University of California Los Angeles, United States of America

## Abstract

**Background:**

In Malawi, there has been a return of *Plasmodium falciparum* sensitivity to chloroquine (CQ) since sulfadoxine-pyrimethamine (SP) replaced CQ as first line treatment for uncomplicated malaria. When used for prophylaxis, Amodiaquine (AQ) was associated with agranulocytosis but is considered safe for treatment and is increasingly being used in Africa. Here we compare the efficacy, safety and selection of resistance using SP or CQ+SP or artesunate (ART)+SP or AQ+SP for the treatment of uncomplicated falciparum malaria.

**Methodology and Findings:**

455 children aged 1–5 years were recruited into a double-blinded randomised trial comparing SP to the three combination therapies. Using intention to treat analysis with missing outcomes treated as successes, and without adjustment to distinguish recrudescence from new infections, the day 28 adequate clinical and parasitological response (ACPR) rate for SP was 25%, inferior to each of the three combination therapies (p<0.001). AQ+SP had an ACPR rate of 97%, higher than CQ+SP (81%) and ART+SP (70%), p<0.001. Nineteen children developed a neutropenia of ≤0.5×10^3^ cells/µl by day 14, more commonly after AQ+SP (p = 0.03). The mutation *pfcrt* 76T, associated with CQ resistance, was detected in none of the pre-treatment or post-treatment parasites. The prevalence of the *pfmdr1* 86Y mutation was higher after treatment with AQ+SP than after SP, p = 0.002.

**Conclusions:**

The combination AQ+SP was highly efficacious, despite the low efficacy of SP alone; however, we found evidence that AQ may exert selective pressure for resistance associated mutations many weeks after treatment. This study confirms the return of CQ sensitivity in Malawi and importantly, shows no evidence of the re-emergence of *pfcrt* 76T after treatment with CQ or AQ. Given the safety record of AQ when used as a prophylaxis, our observations of marked falls in neutrophil counts in the AQ+SP group requires further scrutiny.

**Trial Registration:**

Controlled-Trials.com ISRCTN22075368

## Introduction

Malaria is responsible for around 1 million deaths annually in sub-Saharan Africa, especially in young children. Attempts to control malaria have been hampered because of resistance in *Plasmodium falciparum* to the most commonly used drugs, chloroquine (CQ) and sulfadoxine-pyrimethamine (SP). Malawi switched from CQ to SP as first line treatment for uncomplicated falciparum malaria in 1993, the first country in sub Saharan Africa to do so. SP failure rates have risen since, to day 28 parasitological failure rates of 73% in 2002 [1] and 79% in 2005 [2]. In the light of this declining efficacy, Malawi is changing its first line therapy again. The World Health Organisation (WHO) recommends the use of combination therapies of two or more drugs that target different pathways to overcome resistance-in particular, artemisinin combination therapies (ACTs) [3]. Many countries in sub Saharan Africa have adopted ACTs though in several of these countries, this policy has yet to be implemented [4].

The combination amodiaquine (AQ) plus SP has been shown in some African countries to have efficacy similar to ACTs [5]. AQ is a 4-aminoquinoline like CQ but remains efficacious in many areas with substantial CQ resistance [6], [7]. When taken weekly to prevent malaria, amodiaquine was associated with agranulocytosis and hepatitis [8], [9]; large scale safety data on AQ as a malaria treatment is lacking, but its safety is considered to be acceptable [10]. AQ is increasingly being used in Africa, usually in combination with artesunate (ART). Chloroquine (CQ) plus SP has been less efficacious than AQ+SP in trials in Africa owing to widespread CQ resistance. CQ-resistant parasites carry the 76T mutation on the gene *pfcrt* [11]. In Malawi, the prevalence of this marker has fallen since the switch from CQ to SP (85% in 1992, 0% in 2001) [12]. In addition there has been a fall in the prevalence of mutations in the gene *pfmdr1*; the association of these mutations with CQ resistance is less clear. Recently a return of in vivo CQ sensitivity, predicted by these molecular changes, has been reported from Malawi [2].

Here we report the results of a randomised double-blind clinical trial comparing the efficacy and safety of 3 SP based combination therapies, CQ+SP, artesunate (ART)+SP and AQ+SP with that of SP alone for the treatment of uncomplicated malaria in young children in Malawi. At the time of the study, SP monotherapy was the treatment recommended for uncomplicated malaria by the Malawi National Malaria control committee. In addition, we compared the selection of resistance associated mutations in the parasite genes *dhfr* and *dhps* (associated with SP resistance [13]) and *pfcrt* and *pfmdr1* (associated with AQ and CQ resistance).

## Methods

The protocol for this trial and supporting CONSORT checklist are available as supporting information; see Checklist S1 and Protocol S1

### Participants

The study was based at Chileka health centre near Blantyre, Malawi, where malaria transmission is perennial, peaking during December to April. Between September 2003 and December 2005, children presenting with an illness suggesting falciparum malaria were screened. Inclusion criteria were: i) age ≥12 and <60 months, ii) weight ≥6 kg, iii) axillary temperature ≥37.5°C, iv) no history of treatment with an antimalarial, cotrimoxazole or a tetracycline antibiotic in the previous week, v) no features suggesting severe malaria or a concomitant illness, vi) haemoglobin ≥5.0 g/dl using Hemocue®, and vii) *P. falciparum* monoinfection with a parasite density between 2000 and 200,000 parasites per µl. Written informed consent was required from the parent of each child recruited. The study protocol was approved by ethics committees of the College of Medicine, University of Malawi and Liverpool School of Tropical Medicine. A data and safety monitoring board and local study monitor were appointed.

### Treatment, Randomisation and Blinding

Children meeting all inclusion criteria on day 0 were recruited and randomised to one of four treatment groups. Randomisation was in blocks of 12 according to an off-site computer-generated code to assign patients equally to the four oral treatment groups: SP (25 mg/kg sulfadoxine and 1.25 mg/kg pyrimethamine as a single dose on day 0)+vitamin C 50 mg tablet (placebo) daily for 3 days; CQ (10 mg/kg on days 0 and 1, and 5 mg/kg on day 2)+SP; ART (4 mg/kg once daily for 3 days)+SP; or AQ (10 mg/kg daily for 3 days)+SP. In the case of children too young to swallow tablets, CQ syrup (50 mg per 5mls) and AQ syrup (50 mg per 5mls) were used (same doses as above). The other study drugs were not available as syrups and were crushed and given on a spoon with water if the child could not swallow a tablet. The different tablets were not identical in appearance or taste. A three-day supply of paracetamol (10 mg/kg) was given.

Each child was given a unique study number, assigned sequentially. A dedicated study ‘drug dispenser’ opened the corresponding randomisation envelope and directly observed all drug doses but was not involved in the assessment of children. All other members of the study team were blinded to the dispensing process and patients were uninformed of their treatment allocation for the duration of the study. Children were observed for 30 minutes after dosing. If the child vomited, a second dose was given. If vomiting occurred a second time, the child was withdrawn and treated with parenteral quinine.

### Classification of Outcomes

Patients were assessed on days 0, 1, 2, 3, 7, 14, 28 and 42 and any other day if unwell. Blood was collected for parasite microscopy, storage on Whatman 3M filter paper and, at specified visits, for determination of the full blood count and biochemical parameters. Clinical outcome was assessed using the 2003 WHO therapeutic efficacy protocol for areas of intense malaria transmission [14]. Participants were withdrawn if they failed to attend for follow up, withdrew consent or took a ‘banned’ drug i.e. all antimalarials, cotrimoxazole, doxycycline, tetracycline, chlorpheniramine and folic acid. Late clinical failures were treated with oral mefloquine (25 mg/kg). Severe malaria was treated with parenteral quinine in hospital.

### Statistical methods

The planned sample size of 100 evaluable patients per treatment arm was calculated to have 90% power to detect the difference between an “adequate clinical and parasitological response” (ACPR) rate of 80% with SP alone and 95% with combination therapies using the 5% significance level for each comparison with SP alone. The primary endpoint was the day 28 ACPR rate and the major analysis strategy for the primary endpoint was intention to treat (ITT). Patients with missing outcomes were all classified as successes in one analysis and then as failures in a separate analysis. Per protocol (PP) analysis was also done using polymerase chain reaction (PCR) corrected data to distinguish recrudescences from reinfections. When PCR analysis indicated that a post-treatment parasitaemia was a reinfection, the outcome was classified as a treatment success on that day, but excluded from subsequent analyses. If PCR was inconclusive, the case was excluded from the analysis.

Secondary endpoints included day 14 and 42 ACPR rates, time to fever resolution (axillary temperature≤37.5°C), time to parasite clearance, change in haemoglobin from day 0 to day 14 and the appearance of gametocytes by day 28 after treatment. We also compared adverse events (AEs) between the treatment groups, including self-reported AEs and laboratory AEs; rises in alanine transferase (ALT), total bilirubin, and creatinine between days 0 and 14.

Data were double entered and validated prior to the analyses. Data analysis was performed using Stata 8. Binomial regression was used to obtain risk differences between treatments and 95% confidence intervals. Fisher's exact p-values were reported. Tests of significance were performed using the 5% level to infer significance for the planned analyses. Pair wise comparisons between combination therapies were not planned and in these comparisons we adjusted the significance level to 1.7% (i.e. p<0.017) using Bonferroni's approach.

To look for evidence of selection of resistance mutations in the genes *dhfr*, *dhps*, *pfcrt* and *pfmdr1*, we compared the prevalence of resistance mutations in these genes before and after treatment, both within each of the four different treatment groups and between the four treatment groups.

### Laboratory methods

Blood films were stained with Fields stain and parasite densities estimated from thick films by counting the number of parasites per 200 white blood cells (WBC) assuming a total count of 8000/µl. These parasite counts and haemoglobin (Hb) estimates using Hemocue® were used for screening purposes. In addition, on days 0 and 14, the full blood count was measured using a Beckman Coulter HMX and plasma ALT, total bilirubin and creatinine using a Vitros DTII dry biochemistry analyser. The actual WBC count from the coulter was subsequently used to calculate an accurate parasite count for the analyses. The presence or absence of gametocytes was noted on each blood film.

Parasite DNA was extracted from dried blood on filter paper. A nested PCR was used to distinguish recrudescent infections from new infections in all patients with parasitaemias appearing from day 12 onwards. The *msp2* gene was amplified and size polymorphisms identified by gel electrophoresis using previously described methods [15]. Parasites were classified as recrudescent if they shared any of the bands that were present on day 0 and as reinfections if they had no bands in common. Parasites that appeared before day 12, having initially disappeared, were assumed to be recrudescent. Nested PCR followed by mutation-specific restriction enzyme digestion was used to determine the prevalence of different alleles in the *dhfr*, *dhps*, *pfcrt* and *pfmdr1* genes in day 0 parasites and parasites appearing at any time from day 12 onwards after treatment. A detailed description of these techniques is available at http://www.medschool.umaryland.edu/cvd/plowe.asp. We analysed the *dhfr* gene for polymorphisms at codons 51, 59, 108 and 164; *dhps* codons 437, 540 and 581; *pfcrt* codon 76; and *pfmdr1* codons 86 and 1246. Samples were analysed in a blinded fashion with respect to the treatment groups.

## Results

### Recruitment and Participant flow

**Figure 1:**
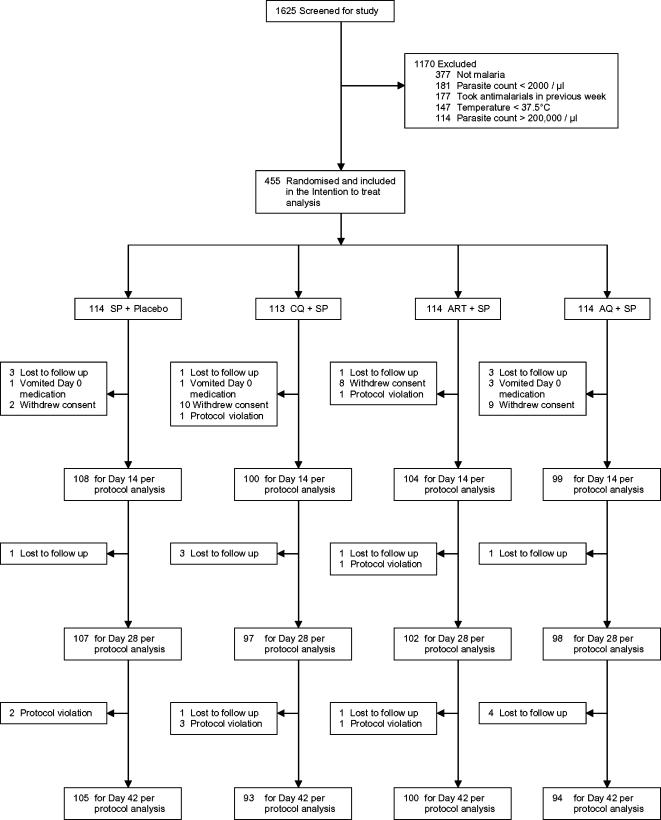
Study Profile

We screened 1625 children and 455 met all inclusion criteria and were enrolled. Baseline characteristics are shown in table 1 and the study profile in figure 1. By day 14, 44 (9.7%) children had been withdrawn from the study, 51 (11.2%) by day 28 and 63 (13.8%) by day 42. The reasons for withdrawal are summarised in figure 1.

**Table 1:** Summary of Baseline characteristics by treatment group

	SP	CQ+SP	ART+SP	AQ+SP
Number of patients	114	113	114	114
Median age (months), (IQR)	22.6 (17.6)	22.2 (14.0)	21.6 (8.2)	21.0 (14.2)
Number (%) female	62 (54.4%)	54 (47.8%)	51 (44.7%)	56 (49.1%)
Number of days of fever	3.1 (1.8)	2.9 (1.8)	3.1 (1.9)	3.1 (1.7)
Weight (kg)	10.9 (2.2)	10.8 (2.3)	10.8 (2.1)	10.8 (2.4)
Initial temperature (°C)	38.8 (0.9)	38.7 (0.9)	38.8 (0.9)	38.8 (0.9)
Geometric mean (range) parasite count* (per µl)	66,171 (973–301,294)	59,874 (2146–280,720)	36,315 (1077–306,722)	44,356 (1892–288,353)
Parasite count>200,000/µl (%)	10 (8.8%)	16 (14.2%)	9 (7.9%)	13 (11.4%)
Gametocytes seen on Day 0	18 (15.8%)	15 (13.3%)	18 (15.8%)	19 (16.7%)
Haemoglobin** (g/dl)	9.1 (1.7)	9.0 (1.6)	8.9 (1.4)	9.3 (1.6)
White cell count (x10^9^/L)	10.2 (4.3)	10.8 (4.6)	9.9 (4.4)	10.3 (4.7)
Neutrophil count (x10^9^/L)	5.1 (3.6)	5.2 (3.6)	4.1 (2.9)	5.3 (4.1)
Platelet count (x10^9^/L)	149 (91)	165 (93)	163 (100)	162 (103)
Alanine Transferase (IU/L)	19 (13)	18 (17)	18 (16)	22 (19)
Total Bilirubin (mg/dl)	0.97 (0.5)	0.95 (0.7)	0.88 (0.7)	0.85 (0.6)
Creatinine (mg/dl)	0.4 (0.1)	0.4 (0.1)	0.4 (0.1)	0.4 (0.1)

For all, data shows mean (SD) unless otherwise indicated

* Parasite count calculated using coulter HB and WCC

** HB from coulter counter. If not available, the Hemocue HB was used (n = 5)

### Primary Outcome: Treatment Efficacy

Using the ITT approach with missing outcomes treated as successes, the day 28 ACPR rate was lowest with SP alone at 25% and inferior to each of the three SP combination therapies (p<0.001), table 2. AQ+SP had an ACPR rate of 97%, higher than each of CQ+SP and ART+SP (p<0.001). There was no significant difference between CQ+SP and ART+SP.

**Table 2:** Day 28 ACPR rates and differences (95% confidence intervals) between the groups by intention to treat analysis–data not PCR adjusted

Comparison	ITT analysis (Withdrawals counted as successes)			ITT analysis (Withdrawals counted as failures)		
	Success rate	Difference (95% confidence interval)	P-value	Success rate	Difference (95% confidence interval)	P-value
SP	25%			19%		
CQ+SP	81%	56% (45%, 67%)	<0.001	67%	48% (37%, 59%)	<0.001
ART+SP	70%	45% (33%, 56%)	<0.001	60%	40% (29%, 52%)	<0.001
AQ+SP	97%	72% (63%, 80%)	<0.001	84%	64% (54%, 74%)	<0.001
CQ+SP vs. ART+SP		−11% (−22%, −0.2%)	0.063		−8% (−20%, 5%)	0.271
CQ+SP vs. AQ+SP		16% (8%, 24%)	<0.001		16% (5%, 27%)	0.006
ART+SP vs. AQ+SP		27% (18%, 36%)	<0.001		24% (12%, 35%)	<0.001

**Figure 2:**
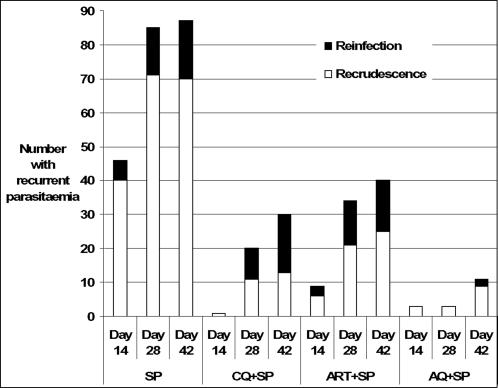
Numbers of recrudescent and new infections in those children with recurrent parasitaemia after treatment.

**Figure 3:**
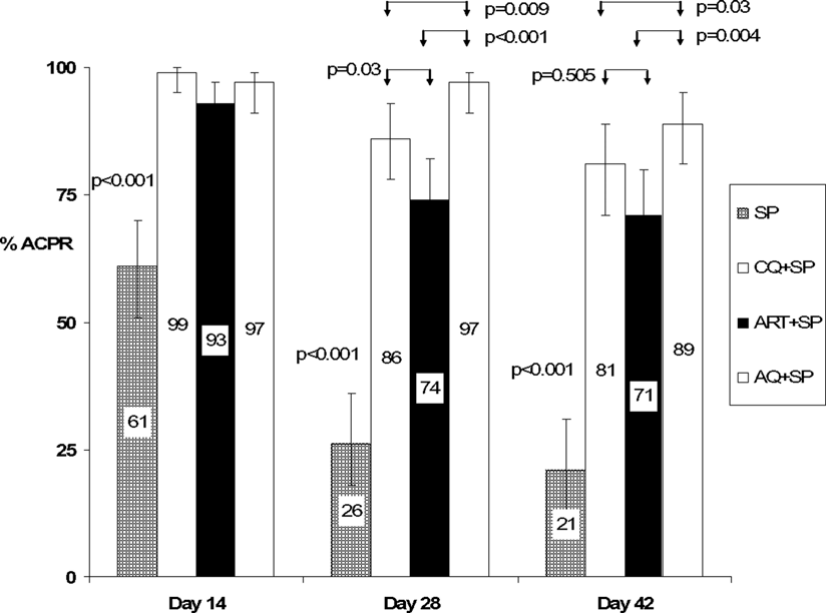
ACPR rates by per protocol analysis, using PCR corrected data.

156 recurrent parasitaemias occurred 12 or more days after treatment. PCR analysis showed that 97 (62%) were recrudescences, 56 (36%) were reinfections and for 3 (2%), the analysis failed. Figure 2 shows the proportions of these in the four treatment groups (recurrent parasitaemias before 12 days were assumed to be due to recrudescence). The PP analysis of these PCR corrected data showed that treatment with SP was inferior to all the SP combinations on day 14, 28 and 42 (p<0.001), figure 3. On day 28, AQ+SP was more efficacious than CQ+SP (p = 0.009) and ART+SP (p<0.001) and on day 42, more efficacious than ART+SP (p = 0.004). The difference between AQ+SP and CQ+SP was not statistically significant on day 42 (p = 0.03) after adjustment using Bonferroni's approach.

### Secondary Outcomes

Ninety-five percent of children had cleared their parasite by day 2 in the ART+SP group compared to 35% for SP, 47% for CQ+SP, and 55% for AQ+SP (p<0.001 for each comparison with AQ+SP). By days 3 and 7, there were no differences between the three combination therapies and they were all superior to SP alone, p = 0.005. In the SP group, there was no association between the day 0 parasitaemia and time to parasite clearance or between day 0 parasitaemia and clinical outcome. Fever resolution was slower with SP alone; the percentage of children who still had fever on day 1 were 18% for SP, 5% for CQ+SP, 6% for ART+SP and 5% for AQ+SP (p<0.008 for each comparison with SP).

Mean haemoglobin concentration rose in all treatment groups. Compared to SP alone, the adjusted mean on day 14 was greater after CQ+SP (p = 0.03) and AQ+SP (p = 0.002) but not after ART+SP (p = 0.81). Gametocytes were present on day 0 in 73 (16%) children, table 1. There were no differences between the groups in the percentage of children with gametocytes on day 28; 4% after SP, 7% after CQ+SP, 5% after ART+SP and 7% after AQ+SP.

### Safety and Tolerability

284 clinical AEs were reported in 185 children. Cough was commonest, making up 45% of all AEs. Compared to SP alone, cough was more commonly reported after ART+SP, p = 0.04. No other statistically significant differences were found. There were 8 serious adverse events (SAEs) in the study with no more than 4 in any treatment group. There were no deaths. The SAEs included 2 cases of pneumonia requiring intravenous antibiotics, 1 child with gastroenteritis requiring intravenous fluids and 5 treatment failures requiring hospitalisation for intravenous quinine. For two of these children, this was due to the occurrence of seizures shortly after receiving their medication on day 0 (1 AQ+SP, 1 CQ+SP), and 1 child had persisted vomiting on day 1 and was unable to continue oral treatment (AQ+SP). The other two children had early treatment failures (1 SP, 1 AQ+SP), and were too unwell to continue oral therapy.

Neutrophil counts fell after treatment in all treatment groups but the proportion of children with neutrophil counts of ≤0.5×10^3^/µl on day 14 (having been ≥1.0×10^3^/µl on recruitment) was greatest in the AQ+SP group (11.5%, n = 7), higher than the SP alone group (1.5%, n = 1), p = 0.03. For 17 of the 19 children in which this low neutrophil count was observed, a repeat sample between day 28 and 42 showed neutrophils ≥1.0×10^3^/µl. For the remaining 2 children no further sample was obtained. We noted no evidence of any ill effects due to these transient low neutrophil counts.

Two children in the AQ+SP treatment group developed plasma ALT levels on day 14 greater than three times the upper limit of normal having been in the normal range (15–45 U/L) on recruitment. The possible association of this adverse effect with AQ+SP was not significant, p = 0.24. One of these children had a day 14 ALT of 1540 U/L with a total bilirubin of 1.1 mg/dl (0.1–1.4 mg/dl). By day 42 the ALT had returned to normal. The other child had a day 14 ALT of 473 U/L with a total bilirubin of 2.2 mg/dl. No further samples were collected for analysis. Both children were well and completed 42 days of follow up and both had day 14 neutrophil counts ≥1.0×10^3^/µl.

### Selection of Resistance

The resistance mutations *pfcrt* 76T, *dhfr* 164L and *dhps* 581G were detected in none of the pre-treatment (n = 244, 155 and 134 respectively) or post-treatment (n = 151,145 and 95) infections. *Pfmdr1* 86N (wild type) parasites were present in 219 out of 244 (89.8%) of pre-treatment infections and in 88.1% (134 out of 152) post-treatment infections. Within each treatment group, there was no difference in the proportions of wild and mutant alleles before and after treatment. When comparing between treatment groups, the proportions of *pfmdr1* 86Y (mutant) in the post treatment AQ+SP group (in 4 recrudescences and 1 new infection) was higher than in the SP post-treatment group, p = 0.002. *Pfmdr1* 1246D (wild type) parasites were present in 224 out of 231 (97%) of pre-treatment infections and in 94.5% (86 out of 91) post-treatment infections. This difference was not significant, and there were no differences within or between the four different treatment groups.

The prevalence of *dhfr* mutant alleles was very high in pre-treatment parasites; 99% (156/157) were 108N, 99.5% (254/255) were 51I and 96% (357/371) were 59R. *Dhps* mutants were also common pre-treatment; 97% (33/34) were 437G and 95.1% (355/373) were 540E. Pre-treatment, 91.6% (339 out of 370) of parasites were “quintuple” mutants (all mutant alleles at *dhfr* codons 51, 59 and 108 and *dhps* codons 437 and 540) and post-treatment, this figure was 92% (139/151). Paired analysis of parasites, pre and post treatment, showed no significant differences within or between the treatment groups.

## Discussion

### Interpretation

Ten years after the introduction of SP as first line treatment for uncomplicated malaria in Malawi the day 28 ACPR rate lies below 30%. Over 90% of pre-treatment parasites were “quintuple” mutants; this genotype has been shown to be strongly predictive of SP failure in young children in Malawi [16]. Malawi is in the process of implementing its new treatment policy of artemether-lumefantrine and our data support the decision to abandon SP. This artemisinin-based combination therapy is highly effective and costs around $1 for an adult course. However, it is a relatively complex regime, 6 doses over 3 days with food, and until recently in Africa, supplies of the drug have not matched the huge demand.

Low cost alternatives to artemether-lumefantrine might have been attractive to the Malawi authorities and AQ+SP was one of the combinations considered. In most countries in Africa, antimalarials are obtained without prescription and often taken unnecessarily [17]. In the era of ACTs, such unregulated use will be hard to sustain. Four other fixed ratio ACTs are in development, AQ plus ART is now available in Africa, chlorproguanil-dapsone plus ART has finished phase III testing, and will be submitted to the regulatory authorities in 2008, and piperaquine plus dihyroartemisinin and pyronaridine plus ART are well into phase III trials. Each of these is likely to be cheaper and more practicable to use than artemether-lumefantrine but still likely to be more expensive that AQ+SP or CQ+SP.

This study confirms the return of CQ sensitivity in Malawi and importantly, shows no evidence of the re-emergence of *pfcrt* 76T after treatment with CQ or AQ based combinations. The CQ+SP day 28 PP ACPR rate was 86%, lower than the rate of 99% reported from another randomised study in 2005, also in Blantyre, in which CQ was used as a monotherapy in 80 children with uncomplicated malaria [2]. The children in our study were younger, mean age 22.2 vs. 31.2 months, and had higher parasite counts on enrolment, and this may explain some of this difference. Another possible reason, especially in the absence of *pfcrt* 76T mutants, is that some children in this study may have failed to achieve therapeutic CQ concentrations. A pharmacokinetic (PK) study was nested within this study and data on drug levels will be reported elsewhere.

AQ+SP was significantly more efficacious than CQ+SP, ART+SP and SP alone. AQ+SP also appears to have a longer period of post-treatment prophylaxis than the other treatments; reinfection rates on days 14, 28 and 42 were similar after treatment with SP, CQ+SP or ART+SP but with AQ+SP, no reinfections were seen until after day 28 (figure 2). AQ+SP was more efficacious than CQ+SP against a background of 100% *pfcrt* 76 wild type parasites. In vitro, AQ has greater activity against *P. falciparum* than CQ [18], and this greater potency may be evident here. Alternatively, the lower CQ+SP efficacy may have PK basis and data from the PK study will be used to address this. Treatment with AQ+SP was associated with the selection of parasites with the *pfmdr1* 86Y mutation between day 28 and day 42 after treatment.

The combination of ART+SP for 3 days was the least effective of the three combination therapies. This is not surprising given the poor efficacy of SP. As a monotherapy, ART is usually taken for 7 days; a 3-day ACT course is only efficacious when the second drug retains adequate efficacy. The combination of ART+SP has proven ineffective in other sites in Africa with significant background SP resistance [19], [20]. This study highlights the importance of continuing follow up beyond 14 days; ART+SP had a 93% day 14 ACPR rate and had the fastest parasite clearance rate but efficacy by days 28 and 42 was poor.

### Generalizability and Overall evidence

AQ+SP has shown excellent efficacy in several African studies and a recent meta-analysis concluded that the efficacy of AQ+SP in Africa was similar to that of AQ+ART but inferior to artemether-lumefantrine [5].The results of these studies showed considerable variability, in part due to differences in existing background resistance to SP and AQ and also to differences in the transmission intensities at the study sites; AQ+SP was more efficacious in high transmission areas. This is probably due to increased host acquired immunity in these areas and also to the post-treatment prophylactic properties of the combination. AQ is a pro-drug, but its active metabolite desethyl AQ has a half-life of several weeks, similar to chloroquine and sulfadoxine. However, post-treatment prophylaxis comes at a cost; drugs with long terminal elimination phases promote the selection of parasites with resistance-conferring mutations [21]. As the drug concentration falls to sub-therapeutic levels, recrudescent or newly infecting parasites carrying resistance mutations may be preferentially selected. This is especially important in areas of moderate to high transmission where re-infections are common and parasite densities may be high.

We saw evidence of the selection of the *pfmdr1* 86Y mutation after treatment with AQ+SP. This mutation is associated with in vivo AQ resistance [22]. Other studies have demonstrated the selection of *pfmdr1* 86Y and *pfmdr1* 1246Y mutations after treatment with AQ+ART [23], [24], and after treatment with AQ+SP [25]. Studies from Zanzibar and Uganda have demonstrated significant increases in parasites carrying the *pfmdr1* 86N allele after treatment with artemether-lumefantrine [26], [27]. This allele has been associated with decreased lumefantrine sensitivity in vitro [28]. These studies provide evidence that these combination therapies are able to drive the selection of resistance mutations and it will be important to monitor closely their efficacy, especially in high transmission areas, to see whether these genotypic changes translate into treatment failures. Artemisinins have short half lives (measured in hours) while all of the companion drugs (apart from chlorproguanil-dapsone) in ACTs recommend by the WHO or under development have half-lives in the order of weeks. Whether the combination chlorproguanil-dapsone plus ART, in which both component drugs have short (closer matched) half-lives, will prevent this selection process, remains to be seen.

We were unable to detect *dhfr* 164L mutants using conventional PCR. This mutation is associated with failure of chlorproguanil-dapsone. One group, using real time PCR, has reported this mutation in 4 of 85 (4.7%) samples from pregnant women in Blantyre collected in 2003 [29]. We were unable to detect any *dhfr* 164L mutants in 158 samples collected in this study using a modified version of the same method, suggesting that if the mutation is present in this region, it remains extremely rare (Ochong E *et al*, in preparation).

AQ was withdrawn as a prophylaxis against malaria in 1986, because of agranulocytosis (1 in 2,100 subjects) and hepatitis (1 in 15,650) [30]. These AEs have not been reported when AQ has been taken as malaria treatment. Between 1984 and 1987, 20 cases of AQ associated agranulocytosis were reported in the literature in individuals taking AQ for malaria prophylaxis [8], [9], [31]–[34]. The shortest duration of prophylaxis prior to diagnosis of agranulocytosis was 3 weeks and lowest total dose consumed 1.2 grams. The treatment dose for a 70 kg adult is 2.1 grams. Neutrophil counts fell after treatment in all treatment groups in this study but the proportion of children with neutrophil counts of ≤0.5×10^3^/µl on day 14 was higher in the AQ+SP group than the SP alone group, p = 0.03. We also observed marked rises in ALT in 2 children after treatment with AQ+SP though this association was not found to be significant. These observations involve small numbers of children and the study was not powered to detect rare AEs. We do however think that given the track record of safety issues with AQ, these observations require further scrutiny especially as AQ may be used increasingly in African patients who have several episodes of malaria each year.

SP has failed in Malawi after a decade of useful service, and its role in intermittent presumptive therapy in pregnancy (IPT) should also now be re-evaluated. There has been a return of CQ sensitivity in Malawi, and CQ could be considered as a possible replacement for SP in IPT programmes. The potential for CQ to be used as part of combination therapy should be considered with caution unless and until CQ-sensitive parasites predominate throughout the region. Given the extensive cross-border movement of people, it seems likely that CQ-resistant parasites from neighbouring countries would be selected rapidly on redeployment of this drug.

The combination AQ+SP was highly efficacious, despite the low efficacy of SP alone; however, we found evidence that in these circumstances AQ may exert selective pressure for resistance mutations many weeks after treatment. In parts of West Africa where SP and AQ both remain efficacious, the combination AQ+SP could be considered for first line treatment for uncomplicated malaria. The drugs have similar pharmacokinetic profiles and the combination may offer a cheaper, longer lasting and readily available alternative to ACTs, with the benefit of longer post-treatment prophylaxis. AQ+ART is being increasingly used and it will be interesting to see whether this combination, with its mismatched kinetics, can prevent the development of AQ resistance. In addition, it will be important to monitor for potential toxicities of AQ after repeated treatment doses.

## Supporting Information

Checklist S1 CONSORT Checklist
(0.06 MB DOC)

Protocol S1 Original study protocol as submitted to the Malawi Research Ethics Committee for approval
(0.07 MB DOC)
