# Supplementary material for: Sulfadoxine-Pyrimethamine–Based Combinations for Malaria: A Randomised Blinded Trial to Compare Efficacy, Safety and Selection of Resistance in Malawi
Source: PLoS One. 2008 Feb 13;3(2):e1578. doi: 10.1371/journal.pone.0001578 (PMC2229666; doi:10.1371/journal.pone.0001578)
Supplement: Protocol S1 — Original study protocol as submitted to the Malawi Research Ethics Committee for approval (0.07 MB DOC) [file pone.0001578.s002.doc]

Evaluating strategies to delay the emergence of resistance to antimalarial drugs

Investigators Dr David Bell, MRCP(UK)

Mr Standwell Nkhoma, BSc (Univ. Malawi) – for PhD studies

# Supervisors Professor Malcolm Molyneux (MLW, College of Medicine)

Professor Peter Winstanley (University of Liverpool)

Collaborators Dr Chris Nkandala, DHO, Blantyre

Dr Peter Kazembe, National Malaria Control Committee

Prof Jack Wirima, Blantyre, Malawi

Professor Chris Plowe (University of Maryland)

Brief CV of investigator

See separate attachment

Institutions involved

#

# The College of Medicine, University of Malawi

# The Department of Pharmacology, Liverpool University, UK.

- The School of Tropical Medicine, Liverpool University, UK.
- Malaria Section, University of Maryland School of Medicine, USA

Executive Summary

The development of resistance to antimalarial drugs is of huge public health concern. In Malawi, sulphadoxine-pyrimethamine (SP) replaced chloroquine (CQ) in 1993 as the first line treatment for uncomplicated malaria because of high levels of CQ resistance (CQR). However SP-resistance is increasing. **Combination therapy (CT)** is proposed as a means of slowing the development of parasite resistance. Artesunate (ART) is suggested as an ideal drug for an anti-malarial combination. Recently, evidence has become available supporting the use of a non-ART CT as a possible alternative.

- There has been a marked decline in the incidence of *pfcrt* (a molecular marker for CQ resistance) in Malawi, suggesting a possible return to CQ sensitivity.
- Amodiaquine (AQ) in combination with SP has been shown to be efficacious and well tolerated in Uganda in an area of high-level CQ resistance.

This issue is of huge public health importance, as combinations of CQ or AQ plus SP would be considerably more affordable than any form of ‘artemisinin combination therapy’.

This study will use molecular tools to look for evidence that CT (both with and without artemisinin drugs) can slow the emergence of resistance genotypes in the study population based on our current understanding of antifolate and quinoline resistance mechanisms. Four different treatment regimes will be compared in a randomised clinical trial; ART-SP, CQ-SP, AQ-SP and SP alone. The prevalence of known resistance alleles will be compared between parasites present in the original infection and those appearing after treatment and attempts will be made to distinguish recrudescent infections from re-infections. To address the possibility that treatment failure could have a pharmacokinetic basis, I will incorporate a population pharmacokinetic study. I will determine whether re-introduction of CQ in a combination leads to the rapid re-selection of *pfcrt* mutations. Clinical outcomes will be compared and the results reported to the National Malaria Control Committee in Malawi. Recently, several new resistance mutations have been identified in vitro associated with high levels of pyrimethamine resistance. To seek novel mutations in vivo, full sequencing of the known resistance associated genes will be performed on parasites in all cases of treatment failure not predictable by the pharmacokinetic data or parasite genotype.

**Background/Introduction**

In 1993, because of high levels of chloroquine resistance, Malawi switched from using Chloroquine as first line therapy for uncomplicated malaria to sulfadoxine-pyrimethamine. However, SP-resistance is increasing and currently in Malawi about 20% of SP treatments fail (Kazembe P, personal communication).

Combination therapy (CT) of two or more drugs working by different mechanisms is proposed as a means to delay development of resistance to anti-malarial drugs. An ideal combination rapidly reduces parasite biomass leaving only a small residual parasite population exposed to high concentrations of the second drug[[1]](#endnote-2). Artesunate (ART), an artemisinin derivative, causes a rapid reduction in parasite biomass with a parasite reduction ratio in the range of 10^3^-10^5^ (ref. [[2]](#endnote-3)) and would be an ideal drug for an anti-malarial combination. In 1994, the introduction of an ART-mefloquine combination as routine treatment for malaria on the Thai-Burmese border (a low transmission area) improved clinical cure rates and brought about a return of in vitro parasite sensitivity to mefloquine[[3]](#endnote-4). In Africa, combination therapies, both with and without artemisinins, have been shown to be well tolerated and to have clinical benefit in terms of accelerated cure rate and symptom relief[[4]](#endnote-5) [[5]](#endnote-6)**.**

The development of SP resistance in areas of high transmission is facilitated by its slow elimination permitting a prolonged period during which blood levels of the drugs exert selection pressure for resistance[[6]](#endnote-7). Both sulfadoxine (Sd) and pyrimethamine (Pm) exert their anti-malarial effect by interrupting *P.falciparum* folate metabolism; they are always used in synergistic combination. Pm competes for the active site of the parasite enzyme dihydrofolate reductase (DHFR) and Sd competes for dihydropteroate synthase (DHPS). Four point mutations at codons 108, 51, 59 and 164 on DHFR are well established for their ability to decrease parasite sensitivity to Pm and are associated with in vivo resistance[[7]](#endnote-8). DHPS gene point mutations at codons 437, 436, 581, 540 and 613 are associated with a decreased susceptibility to Sd, although the relevance of sulfadoxine resistance to clinical response after SP is less clear[[8]](#endnote-9). SP use is associated with the disappearance of fully drug-sensitive parasites and a significant increase in the number of point mutations conferring resistance in both the DHFR and DHPS genes in subsequent parasitaemias (re-infections or recrudescents) occurring within 42 days of treatment[[9]](#endnote-10).

Novel DHFR mutations may also exist in vivo that confer increased resistance. A yeast-based system with the DHFR gene inserted has allowed the screening of over 1000 functional DHFR alleles to determine their sensitivity to Pm. New mutations on the DHFR domain have been identified that increase resistance to Pm by 10-100 fold in vitro[[10]](#endnote-11). This work raises the possibility that other novel resistance mutations are present contributing to in vivo resistance.

Mutations in the gene *pfcrt*, which encodes a transmembrane protein in the digestive vacuole of the malaria parasite, have recently been demonstrated as being strongly associated with CQ resistance (CQR). Several mutations have been identified but the K76T mutation appears critical. As a molecular marker for CQR in vivo, this mutation is almost 100% sensitive and 40–50% specific[[11]](#endnote-12) [[12]](#endnote-13), and it has been shown to confer resistance in genetic transformation studies[[13]](#endnote-14). Another gene, pfmdr1, encoding Pgh-1, a food vacuole glycoprotein, has been suggested as also having a role in modulating CQR[[14]](#endnote-15). Many polymorphisms of this gene have been described but the N86Y mutation has been the most investigated in Africa. Some studies have shown associations between this mutation and in vivo CQR but others have not[[15]](#endnote-16).

Amodiaquine (AQ), another 4-aminoquinolone, was widely used in the past to treat and prevent malaria but concerns about its safety led to its withdrawal as a first line agent. In the face of increasing levels of CQ and SP resistance, the use of AQ is increasingly being proposed in Africa. AQ has superior antimalarial activity to CQ and cross-resistance to CQ although present (especially to the active desethyl metabolite of AQ) is small[[16]](#endnote-17). A review of AQ treatment for uncomplicated malaria did not identify any additional side-effects or toxicity when compared to CQ or SP treatment[[17]](#endnote-18).

**Rationale/Justification**

In parts of East Africa, high levels of CQ resistance may limit its effectiveness in combination therapies. Although CQ resistance has been thought to be common and stable in Malawi, recent data open this to question. The chloroquine resistance conferring mutation K76T in the gene *pfcrt* had a prevalence of 85% in Malawi in 1992 shortly before SP replaced CQ as first line therapy. Recent work has shown that this prevalence has fallen to around ~13%, suggesting a return to CQ sensitivity (Plowe CV; personal communication)*.* Recently, over 50 Malawian adults with asymptomatic parasitaemias were treated with CQ alone and all but one (whose parasitaemia could be due to re-infection) cleared their parasitaemia (Plowe CV; personal communication). Another study using parasites from Malawian children in 1998 noted a significant reduction in the proportion of CQ resistant isolates on in vitro testing compared to a similar study in 1988, suggesting a possible recovery of CQ sensitivity after long-term absence of drug pressure[[18]](#endnote-19).

There is merit in examining the possibility that, in Malawi, CQ in combination with another drug such as SP might offer clinical benefit as well as a reduction in the rate of development of SP resistance. Reintroduction of CQ as a single agent is likely to re-select the *pfcrt* mutation but, in combination with SP, selection of CQ resistance may also be prevented or delayed. The involvement of *pfcrt* in parasite susceptibility to AQ has not been addressed and the role of non-K76T *pfcrt* mutations in determining CQR needs further evaluation. The relationship between *pfmdr1* and clinical outcome remains to be clarified.

ART has a very short half-life (~60mins) and rapidly leaves the second drug in a combination unprotected. From a pharmacokinetic point of view, closer matching of the elimination half-lives of drugs in a combination may reduce the selective pressure for resistance in high transmission areas. Both CQ and AQ have pharmacokinetic profiles closer to SP than ART and also achieve rapid reductions in circulating parasitaemia with parasite reduction ratios in the range of 10^2^-10^4^ (ref.2). If the theoretical arguments in favour of CT therapy are accepted, AQ in combination with SP should be an effective and affordable alternative for Africa. In support of this a combination of AQ with SP has recently been shown in Uganda to be highly effective with no increase in adverse effects reported[[19]](#endnote-20).

In comparing malaria treatments it is important to distinguish whether parasites appearing after treatment are due to re-infection or recrudescence. In this study, polymorphic loci (microsatellite markers) on the *P. falciparum* genome will be compared pre and post treatment to determine as accurately as possible whether parasites are recrudescent or new. One protocol characterising twelve polymorphic loci has been previously described[[20]](#endnote-21). The use of microsatellite markers will enable the study to be controlled for the potential complications of non-clonal infections. To address whether treatment failure can be explained by inadequate treatment, drug plasma concentrations will be measured in all patients and population pharmacokinetic models established. Novel DHFR mutations conferring increased *in vivo* resistance will be looked for in patients with treatment failure despite adequate drug concentrations and whose parasite genotype suggests sensitivity.

# Objectives of the study

In the treatment of uncomplicated *P falciparum* malaria, the following hypotheses will be tested: -

1. Compared to the standard regimen of SP alone, the addition of CQ or AQ or ART to SP results in:

- Improved clinical outcome in this clinical setting.
- Decreased selection of resistance mutations in the dihydrofolate reductase (DHFR), dihydropteroate synthetase (DHPS), *pfcrt* and *pfmdr1* genes in new and recrudescent infections occurring up to 42 days after treatment.

1. Use of CQ in combination with SP does not lead to a rapid re-selection of *pfcrt* mutations.
2. Treatment failures not predictable by genotype or pharmacokinetic data are associated with novel DHFR, DHPS, *pfcrt* and *pfmdr1* mutations.

**Methods**

### Type of study

A randomised clinical trial comparing SP plus placebo to SP plus one of CQ, AQ and ART over a standard 3 day regime. All patients will be followed up using a modified version of the WHO in-vivo protocol, the clinical end-points being acceptable clinical response (ACR), early treatment failure (ETF) and late treatment failures (LTF)[[21]](#endnote-22). Children, who become unwell from days 15 to 42 post treatment, will remain under follow up in the study. Children who develop severe malaria while in the study will receive quinine treatment as is standard in Malawi. Children with treatment failure but no severe symptoms will be treated with *Co-Artem* (Artemether plus lumefantrine). The tolerability of the four regimes will be assessed by symptom enquiries from the patients. Laboratory and clinical staff will be blinded to the treatment groups.

Place of study

The children will be recruited and followed up from Chilomoni Health Centre, which is situated in a peri-urban area of Blantyre.

Study population

Children aged 12 to 60 months. Entry criteria will include a pure mono-infection (on microscopic grounds) of *P falciparum* with parasitaemias ranging from 20,000 to 200,000 μL^-1^ and no clinical feature suggestive of severe malaria[[22]](#endnote-23).

Study period

A continuous 2-year period in Malawi starting in March 2003.

Sample size

500 children will be recruited. The sample size has been determined to detect the difference between an ACR rate of 80% with SP alone and 95% with CQ-SP using p=0.05 and a type 2 error of 10%. Each arm of the trial will have 125 children, which allows for a 25% loss to follow up rate. To obtain rich data for the pharmacokinetic study, a further 12 older children will be recruited for each trial arm, and treated unblinded.

Data collection

All patients will have a Case Record Form (CRF) completed: this will include details of a standardised history and examination, side-effect enquiries and the results of investigations. The CRF will be up-dated at every subsequent consultation.

A population pharmacokinetic (PK) model will be developed for each drug in the trial, Pm, Sd, CQ and AQ. Because the trial is blinded, it is necessary to collect four 5ml venous samples from each child to be sure of collecting adequate data on all four of the treatment regimes. The first sample will be taken within 3 hours of the initial drug dose, the second, third and fourth at random times on days 3, 7 and 14 when the children attend the clinic for review. To obtain rich PK data, a further 12 older children will be recruited for each trial arm, and treated unblinded. Seven 2ml venous samples will be collected following a defined schedule depending on which treatment regime they have received. Pm, Sd, CQ, desethyl-CQ, AQ and desethyl-AQ plasma concentrations will be measured on these samples by high performance liquid chromatography (HPLC) at the Wellcome Trust Research Laboratory in Blantyre. ART and dihydro-ART concentrations will be determined in Liverpool on stored samples by LC/Mass Spectrometry.

An initial blood film will be prepared from a finger-prick sample on recruitment. Subsequent blood films will be prepared from the venous samples collected as detailed above. Haemoglobin concentration, white cell count, platelet count, bilirubin, gamma-glutamyl transpeptidase and creatinine will be determined from the venous samples collected on days 0 and 14. Gametocyte count on day 7 will be documented on all patients.

PCR followed by allele-specific restriction digestion for known DHFR, DHPS, *pfmdr1* and *pfcrt* markers of CQ, Sd and Pm resistance will be completed on all pre treatment parasites and on all parasites appearing up to 42 days post treatment. The protocols for these analyses are described previously[[23]](#endnote-24)13 [[24]](#endnote-25). The genotype of treatment failures and parasites appearing between days 14 to 42 from each of the treatment groups will compared. Microsatellite markers on the *P. falciparum* genome will be analysed by PCR using protocols developed by Chris Plowe’s group (a collaborator on this proposal) to try to distinguish re-infection from recrudescence.

Full DHFR, DHPS, *pfcrt* and *pfmdr1* gene sequencing will be done on all parasites which appear up to 42 days post-treatment despite adequate plasma drug concentrations (judged using the population PK models), and in whom the genotype suggests sensitivity. By these methods I expect to be able to identify novel DHFR, DHPS and *pfcrt* mutations that may arise conferring resistance. The gene sequencing will be performed in collaboration with the University of Maryland sequencing facility.

Using the genotype and parasite clearance / reappearance data a PK / Pharmacodynamic model will be established to establish the interplay between parasite genotype / kill and PK.

###

Data management/analysis

A Data and Safety Management Board with a written constitution will be established at the start of the trial.

**Presentation and dissemination of results**

The end results of the trial will be reported to the National Malaria Control Committee in Malawi, presented at the Annual College of Medicine Research Dissemination Day, and submitted for publication internationally. Results that have a bearing on policy in Malawi will be submitted to the Malawi Medical Journal.

**Ethical considerations**

Patients shall be recruited on presentation to the clinic. They will be offered assessment by the study doctor / clinical officer and if assessment is permitted, and the patient fulfils the entry criteria, written consent will be sought on approved forms. The consent form and patient information sheet will be available in both English and the local language. Since this is a study conducted in minors, the relatives or guardians of the subjects, and if appropriate the subjects themselves, will be given ample opportunity to enquire about details of the study. If the relative or guardian is unable to write their signature then a thumb-print will be used in the presence of a witness. A screening record will be maintained at each site to log all patients who where considered as potential patients but where excluded.

**References**

1. White NJ and Olliaro PL. Strategies for the prevention of antimalarial drug resistance: Rationale for combination chemotherapy for malaria. *Parasitology Today* 1996; 12 (10): 399-401. [↑](#endnote-ref-2)
2. White NJ. Assessment of the pharmacodynamic properties of antimalarial drugs in vivo.

   *Antimicrob. Agents and Chemother.* 1997; 41 (7): 1413-1422. [↑](#endnote-ref-3)
3. Nosten F, van Vugt M, Luxemburger C, Thway K, Brockman A, McGready R, Ter Kuille F, Looareesuwan S and White N. Effect of artesunate – mefloquine combination on the incidence of *Plasmodium falciparum* malaria and mefloquine resitance in western Thailand: a prospective study. *Lancet* 2000; 356: 297 – 302. [↑](#endnote-ref-4)
4. McIntosh HM and Greenwood BM. Chloroquine or Amodiaquine combined with sulphadoxine-pyrimethamine as a treatment for uncomplicated malaria- a systematic review. *Annals of Trop Med Parasit*  1998; 3: 265-270. [↑](#endnote-ref-5)
5. Von Seidlein L, Milligan P, Pinder M, Bojang K, Anyalebechi C, Gosling R, Coleman R, Ude JI, Sadiq A, Duraisingh M, Warhurst D, Alloueche A, Targett G, McAdam K, Greenwood K, Walraven G and Doherty T. Efficacy of artesunate plus pyrimethamine-sulphadoxine for uncomplicated malaria in Gambian children: a double blind, randomised, controlled trial. *Lancet* 2000; 355: 352 – 57 [↑](#endnote-ref-6)
6. Watkins WM and Mosobo M. Treatment of *Plasmodium falciparum* malaria with pyrimethamine-sulphadoxine: selective pressure for resistance is a function of long elimination half-life. *Trans Roy Soc Trop Med Hyg.* 1993; 87: 75-78. [↑](#endnote-ref-7)
7. Nzila AM, Mberu EK, Sulo J, Dayo H, Winstanley PA, Sibley CH and Watkins WM. Towards an understanding of the mechanism of pyrimethamine-sulphadoxine resistance in *Plasmodium falciparum*: Genotyping of Dihydrofolate Reductase and Dihydropteroate synthase of Kenyan Parasites. *Antimicrob. Agents Chemother.* 2000; 44 (4): 991-996. [↑](#endnote-ref-8)
8. Watkins WM, Mberu EK, Winstanley PA and Plowe CV. The efficacy of antifolate combinations in Africa: a predictive model based on pharmacodynamic and pharmacokinetic analyses. *Parasitology Today.* 1997; 13 (12): 459-464. [↑](#endnote-ref-9)
9. Nzila AM, Nduati E, Mberu EK, Sibley CH, Monks SA, Winstanley PA and Watkins WM. Molecular evidence of greater selective pressure for drug resistance exerted by the long-acting antifolate Pyrimethamine/sulphadoxine compared with the shorter-acting Chlorproguanil/Dapsone on Kenyan *Plasmodium falciparum.* *J of Infect Dis.* 2000; 181: 2023 – 8. [↑](#endnote-ref-10)
10. Ferlan JT, Mookherjee S, Okezie IH, Fulgence L and Sibley CH. Mutagenesis of Dihydrofolate reductase from *Plasmodium falciparum*: analysis in *Saccharomyces Cerevisiae* of triple mutant alleles resistant to pyrimethamine or WR99210. *Mol. Biochem. Parasitol*. 2001; 113: 139-150. [↑](#endnote-ref-11)
11. Djimbe A, Doumbo OK, Cortese JF, Kayentao K, Doumbo S, Diourte Y, Dicko A, Su X, Nomura T, Fidock DA, Wellems TE and Plowe CV. A molecular marker for Chloroquine-resistant Falciparum Malaria. *N Engl J Med.* 2001; 344 (4): 257 – 63. [↑](#endnote-ref-12)
12. Dorsey G, Kamya MR, Singh A, Rosenthal PJ. Polymorphisms in the *Plasmodium falciparum* *pfcrt* and *pfmdr-1* genes and clinical response to chloroquine in Kampala, Uganda*. J Infect Dis.* 2001; 183:1417-20. [↑](#endnote-ref-13)
13. Fidock DA, Nomura T, Talley AK, Cooper RA, Dzekunov SM, Ferdig MT, Ursos LMB, Sidhu A, Naude B, Deitsch KW, Su X, Wootton JC, Roepe PD and Wellems TE. Mutations in the *P.Falciparum* digestive vacuole transmembrane protein PFCRT and evidence for their role in chloroquine resistance. *Mol. Cell.* 2000; 6: 861-871. [↑](#endnote-ref-14)
14. Foote SJ, Kyle DE, Martin RK, Oduola AMJ, Forsyth K, Kemp DJ and Cowman AF. Several alleles of the multidrug-resistance gene are closely linked to chloroquine resistance in *Plasmodium falciparum*. *Nature.* 1990; 345: 255-8. [↑](#endnote-ref-15)
15. Wellems TE and Plowe CV. Chloroquine-resistant malaria. *J Infect Dis* 2001; 184(6): 770-6. [↑](#endnote-ref-16)
16. Brasseur P, Guiguemde R, Diallo S, Guiyedi V, Kombila M, Ringwald Pand Olliaro P. Amodiaquine remains effective for treating uncomplicated malaria in West and Central Africa. *Trans R Soc Trop Med Hyg* 1999; 93: 645-50. [↑](#endnote-ref-17)
17. Olliaro P, Nevill C, LeBras J, Ringwald P, Mussano P, Garner P and Brasseur P. Systematic review of amodiaquine treatment in uncomplicated malaria. *Lancet.* 1996; 348: 1196 –201. [↑](#endnote-ref-18)
18. Takechi M, Matsuo M, ziba C, Macheso A, Butao D, Zungu I, Chakanika I and Bustos MDG. Therapeutic efficacy of sulphadoxine/pyrimethamine and susceptibility *in vitro* of *P.falciparum* isolates to sulphadoxine-pyremethamine and other antimalarial drugs in Malawian children. *Trop Med and Int Health*. 2001; 6: 429-434. [↑](#endnote-ref-19)
19. Staedke SG, Kamya MR, Dorsey G, Gasasira A, Ndeezi G, Charlebois ED and Rosenthal PJ. Amodiaquine, sulphadoxine/pyrimethamine, and combination therapy for treatment of uncomplicated falciparum malaria in Kampala, Uganda: a randomised trial. *Lancet*. 2001; 358: 368-74. [↑](#endnote-ref-20)
20. Anderson TJC, Xin-Zhuan SU, Bockarie M, Lagog M and Day KP. Twelve microsatellite markers for the characterisation of *Plasmodium falciparum* from finger-prick blood samples. *Parasitology.* 1999; 119: 113-125. [↑](#endnote-ref-21)
21. World Health Organisation (1996). Assessment of therapeutic efficacy of antimalarial drugs for uncomplicated Falciparum malaria in areas with intense transmission. WHO/MAL/96.1077. [↑](#endnote-ref-22)
22. World Health Organisation (2000). Severe falciparum malaria. *Transactions of the Royal Society of Tropical Medicine and Hygiene*, 94, Supplement 1. [↑](#endnote-ref-23)
23. ^1^ [↑](#endnote-ref-24)
24. Plowe CV, Cortese JF, Doumbo OK et al. Mutations in *Plasmodium falciparum* Dihydrofolate Reductase and Dihydropteroate Synthase and Epidemiologic patterns of Pyrimethamine-sulphadoxine use and resistance. *J of Infect Dis*. 1997; 176: 1590-6. [↑](#endnote-ref-25)
